# Supplementary material for: Cumulative human impacts on global marine fauna highlight risk to biological and functional diversity
Source: PLoS One. 2024 Sep 18;19(9):e0309788. doi: 10.1371/journal.pone.0309788 (PMC11410257; doi:10.1371/journal.pone.0309788)
Supplement: S1 File — (PDF) [file pone.0309788.s001.pdf]

Supporting Information for:

Cumulative human impacts on global marine fauna highlight risk to biological and functional diversity

Casey C. O'Hara[1*], Melanie Frazier[1], Mireia Valle[1,2,3], Nathalie Butt[4,5], Kristin Kaschner[6], Carissa Klein[5], Benjamin S. Halpern[1,7]

Affiliations

1. National Center for Ecological Analysis and Synthesis, University of California - Santa Barbara, Santa Barbara, California, USA
2. AZTI, Marine Research, Basque Research and Technology Alliance (BRTA), Sukarrieta, Spain
3. Basque Centre for Climate Change (BC3), Scientific Campus of the University of the Basque Country (UPV-EHU), Leioa, Spain
4. The Nature Conservancy, Suite 20, Level 1, 88 Tribune Street, South Brisbane, Queensland, Australia
5. The University of Queensland, School of the Environment, Centre for Biodiversity and Conservation Science, Brisbane, Queensland, Australia
6. Department of Biometry and Environmental System Analysis, Albert-Ludwigs-University of Freiburg, Freiburg, Germany
7. Bren School of Environmental Science & Management, University of California - Santa Barbara, Santa Barbara, California, USA

[*]**Correspondence to:** Casey O'Hara, ohara@nceas.ucsb.edu

List of Supporting Materials

Figures S1 – S7

Tables S1 – S4

Supporting Methods

Supporting References (75-98)

Supporting Figures

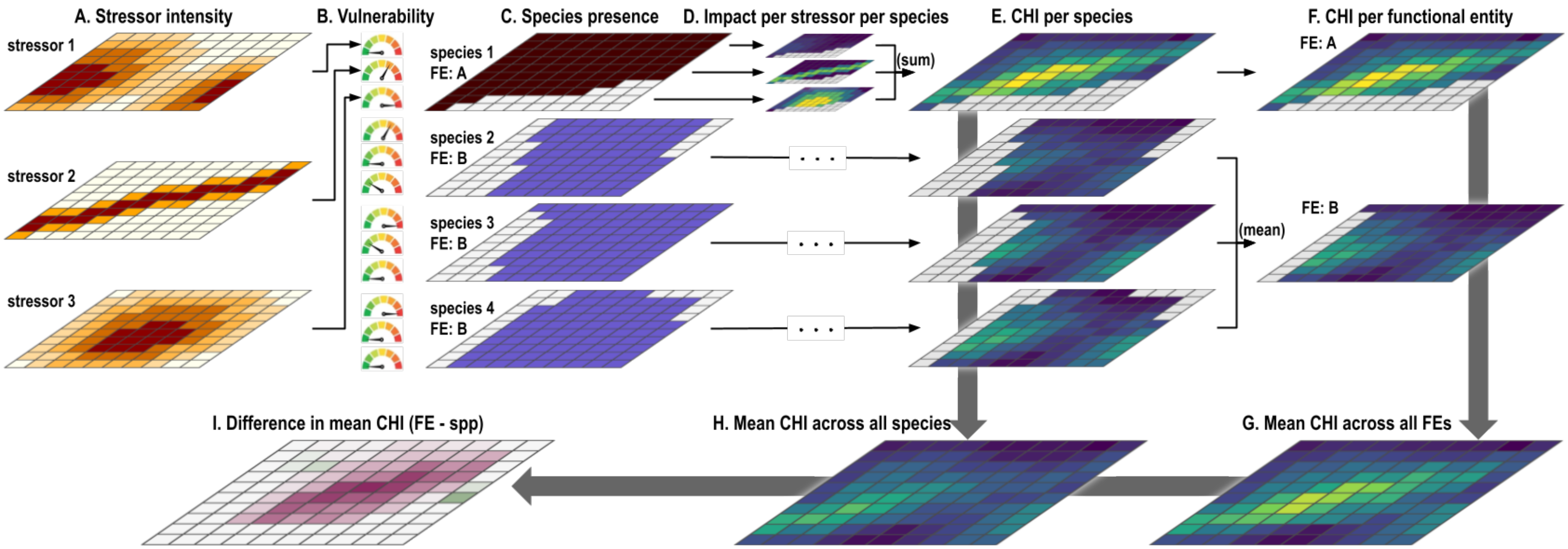

Figure S1. Graphical conceptual summary of cumulative impact calculations. (A) Stressors are mapped as intensity values on a 10 x 10 km grid, with values ranging from 0 to 1. (B) Each species is vulnerable, to different degrees, to each of the stressors, with vulnerability ranging from 0 to 1. (C) The distribution of each species is mapped as present/absent in each 10 x 10 km grid cell. Note the colors represent different functional entities. (D) For each species x stressor combination, an impact map is generated as the product of species presence (C), species vulnerability to the stressor (B), and stressor intensity (A). (E) The cumulative impact to each species is the sum of impacts across all stressors. (F) For the species approach, a mean cumulative human impact is calculated as the average cumulative impact across all species present in each cell. (G) For the functional entity approach, impact values are aggregated to the functional entity level by averaging impact values within each grid cell across all species associated with that functional entity. A functional vulnerability is calculated for each FE/grid cell combination, where a FE with few species is more vulnerable than a FE represented by many species. (H) For the functional entity approach, a mean cumulative human impact is calculated as the average cumulative impact across all functional entities present in each cell, weighted by functional vulnerability. (I) The functional entity impacts and species impacts are compared by taking the difference between the impacts predicted by each method for each grid cell.

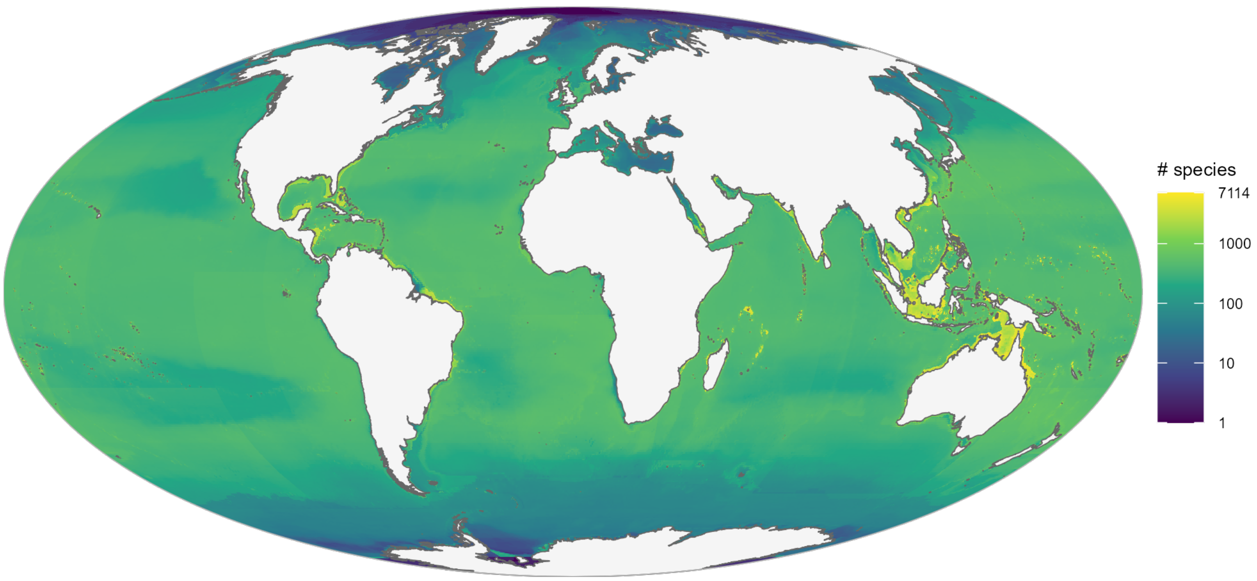

Figure S2. Global pattern of species richness based on 21,159 species range maps included in this assessment.

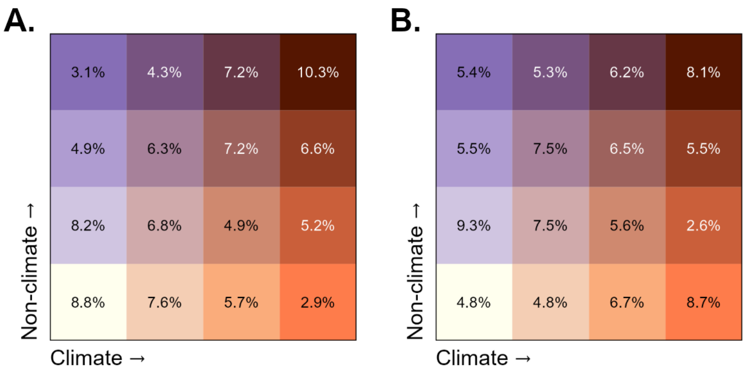

Figure S3. Percent of cells falling into each impact quartile category. (A) Species method. (B) Habitat method. Note that a uniform distribution would result in a value of 6.3% in each impact quartile category.

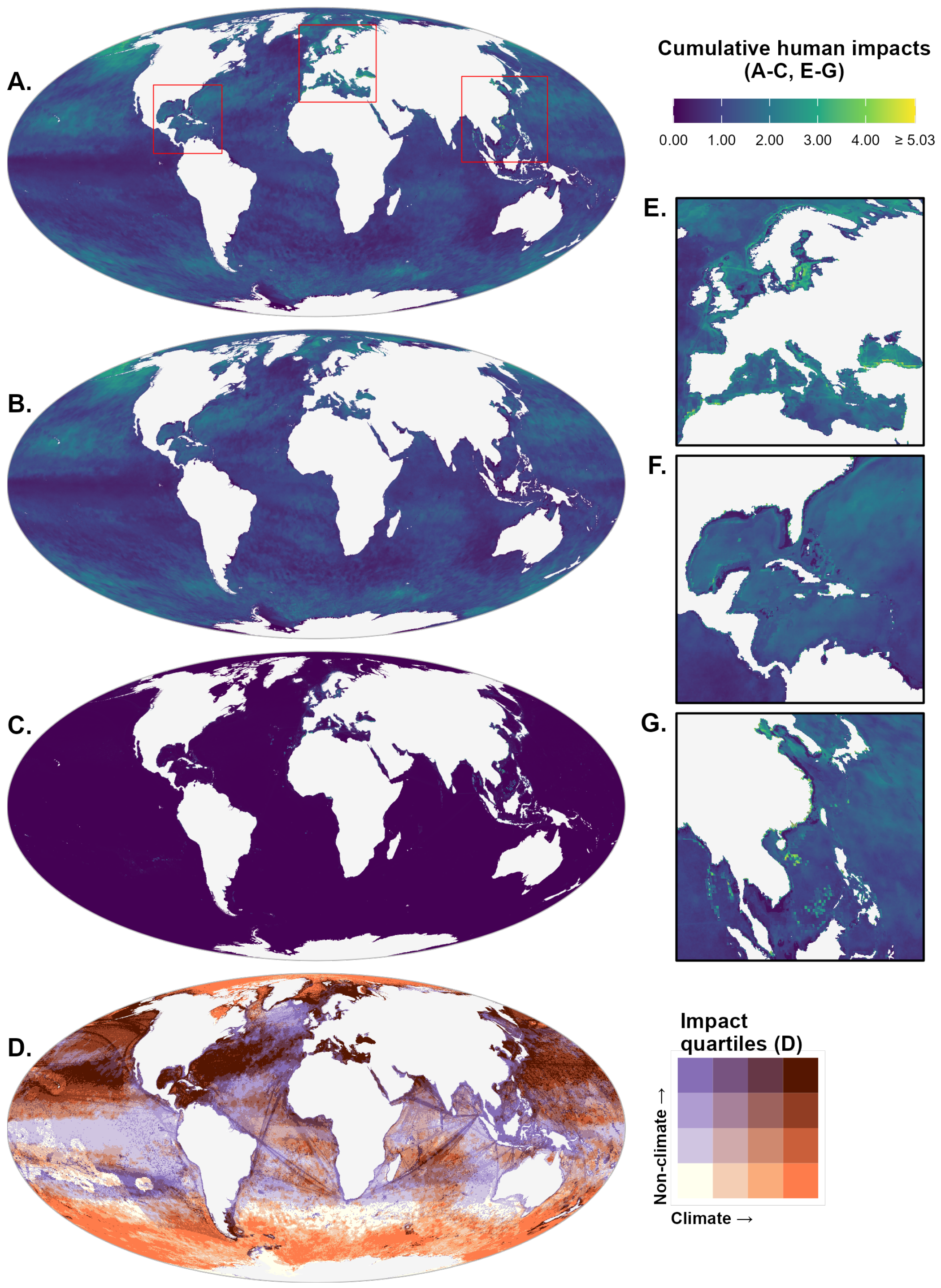

Figure S4. Distribution of modeled risk of impact based on vulnerability and exposure of ecosystem-representative habitats to anthropogenic stressors. (A) Mean cumulative impact across all habitats in each cell, summing across all stressors. (B) Mean cumulative impact across all habitats, summing across all climate-related stressors. (C) Mean cumulative impact across all habitats, summing across all non-climate stressors. (D) Bivariate comparison of distributions of climate impacts (orange) vs. non-climate impacts (purple) by quartile within each stressor group.

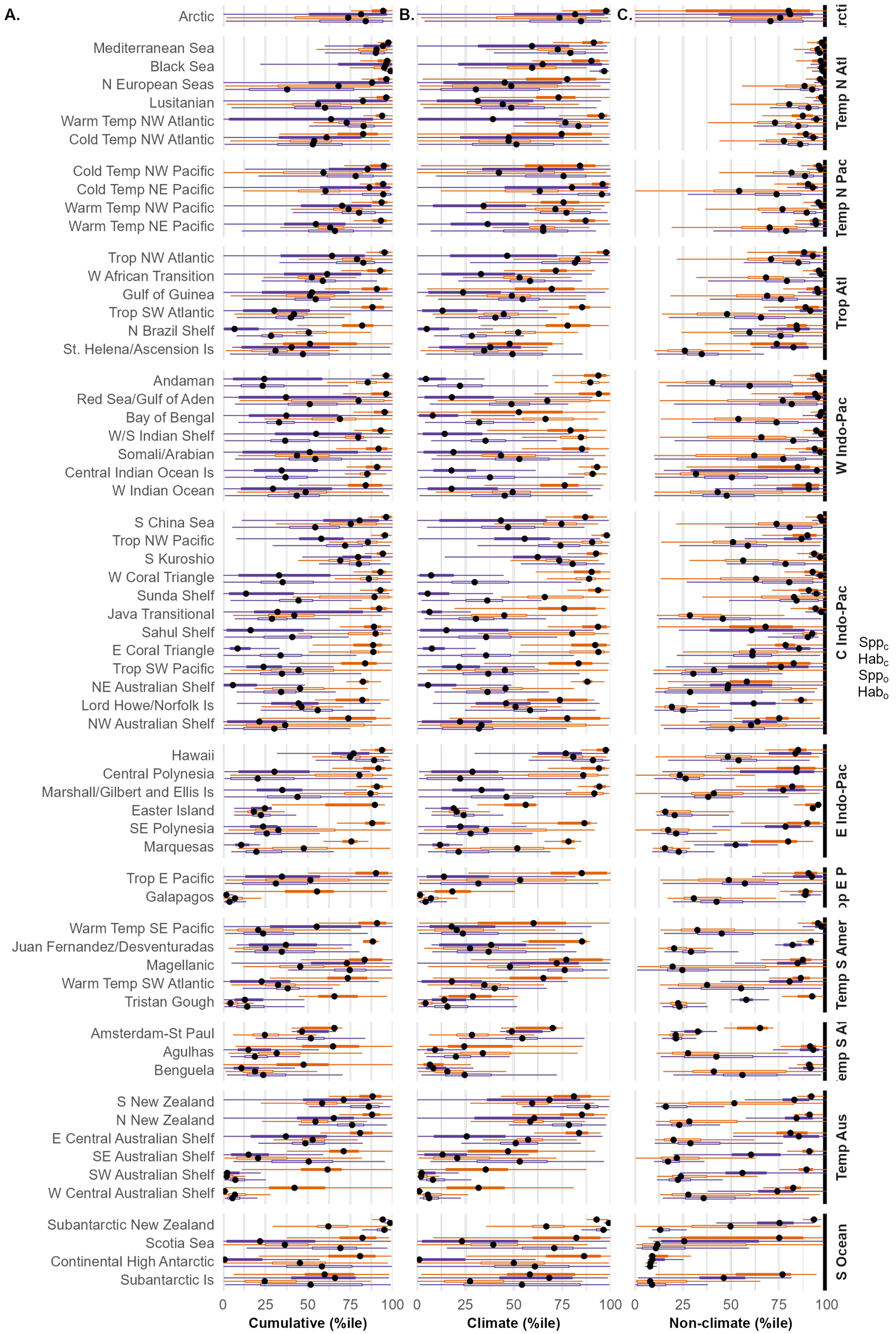

A.
B.
C.
Arctic
Mediterranean Sea
Black Sea
N European Seas
Lusitanian
Warm Temp NW Atlantic
Cold Temp NW Atlantic
Cold Temp NW Pacific
Cold Temp NE Pacific
Warm Temp NW Pacific
Warm Temp NE Pacific
Trop NW Atlantic
W African Transition
Gulf of Guinea
Trop SW Atlantic
N Brazil Shelf
St. Helena/Ascension Is
Andaman
Red Sea/Gulf of Aden
Bay of Bengal
W/S Indian Shelf
Somali/Arabian
Central Indian Ocean Is
W Indian Ocean
S China Sea
Trop NW Pacific
S Kuroshio
W Coral Triangle
Sunda Shelf
Java Transitional
Sahul Shelf
E Coral Triangle
Trop SW Pacific
NE Australian Shelf
Lord Howe/Norfolk Is
NW Australian Shelf
Hawaii
Central Polynesia
Marshall/Gilbert and Ellis Is
Easter Island
SE Polynesia
Marquesas
Trop E Pacific
Galapagos
Warm Temp SE Pacific
Juan Fernandez/Desventuradas
Magellanic
Warm Temp SW Atlantic
Tristan Gough
Amsterdam-St Paul
Agulhas
Benguela
S New Zealand
N New Zealand
E Central Australian Shelf
SE Australian Shelf
SW Australian Shelf
W Central Australian Shelf
Subantarctic New Zealand
Scotia Sea
Continental High Antarctic
Subantarctic Is
0 25 50 75 100
Cumulative (%ile)
Climate (%ile)
Non-climate (%ile)
rcti
Temp N Atl
Temp N Pac
Trop Atl
W Indo-Pac
C Indo-Pac
E Indo-Pac
op E P
Temp S Amer
emp S Af
Temp Aus
S Ocean
Spp_c
Hab_c
Spp_o
Hab_o

Figure S5. Comparison of cumulative, climate, and non-climate stressors by habitat and species methods across 10 km resolution cells within coastal (Spp_c, Hab_c) and oceanic (Spp_o, Hab_o) portions of 62 representative marine ecological provinces, transformed to percentile ranks relative to global distribution within each impact category. Filled point indicates median value; bars represent interquartile range (IQR, quartile Q1 to Q3); whiskers indicate observations 1.5x IQR below (above) Q1 (Q3) of bar. Outliers omitted from plots for clarity. A) Cumulative impacts by species and habitat cumulative impact methods. B) Climate impacts by species and habitat cumulative impact methods. C) Non-climate impacts by species and habitat cumulative impact methods.

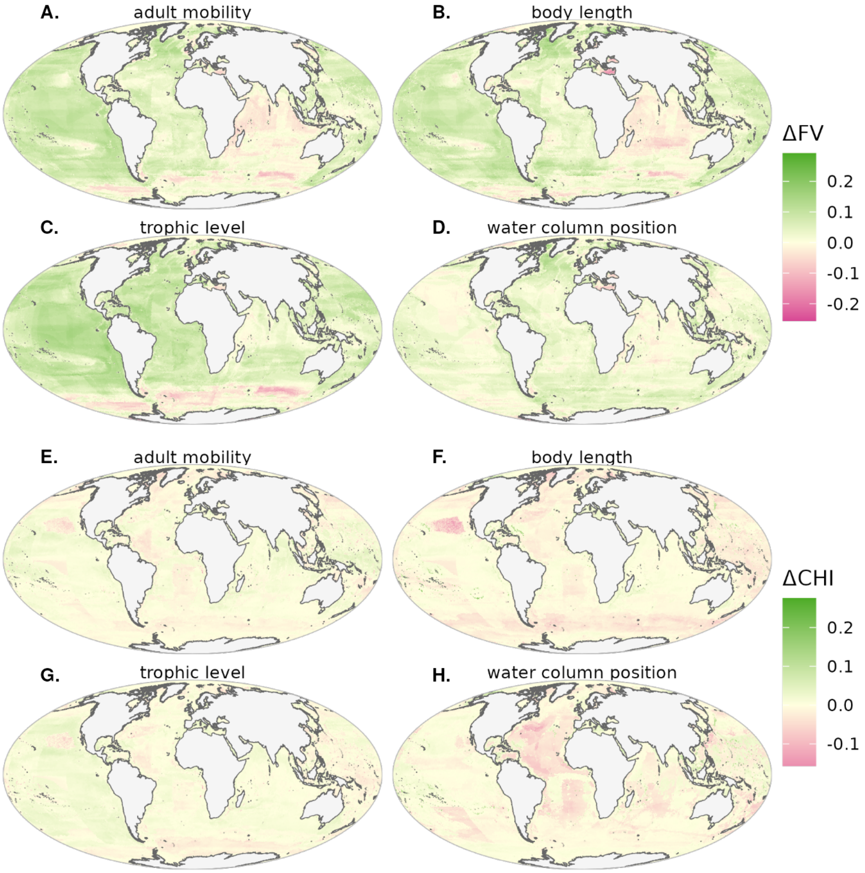

Figure S6. Sensitivity of functional vulnerability (FV) and cumulative human impact (CHI) to trait variation. Each trait was resampled 1000 times, holding other traits constant, and change in functional vulnerability (A-D) and cumulative human impact (E-H) were recalculated according to the Functional Entity approach. Functional vulnerability generally increased slightly (A-D, green tones), as resampling tends to create novel trait combinations not found in nature, leading to more low-membership (and thus high FV) functional entities. The effects of these changes in functional vulnerability on the overall cumulative impact were quite low.

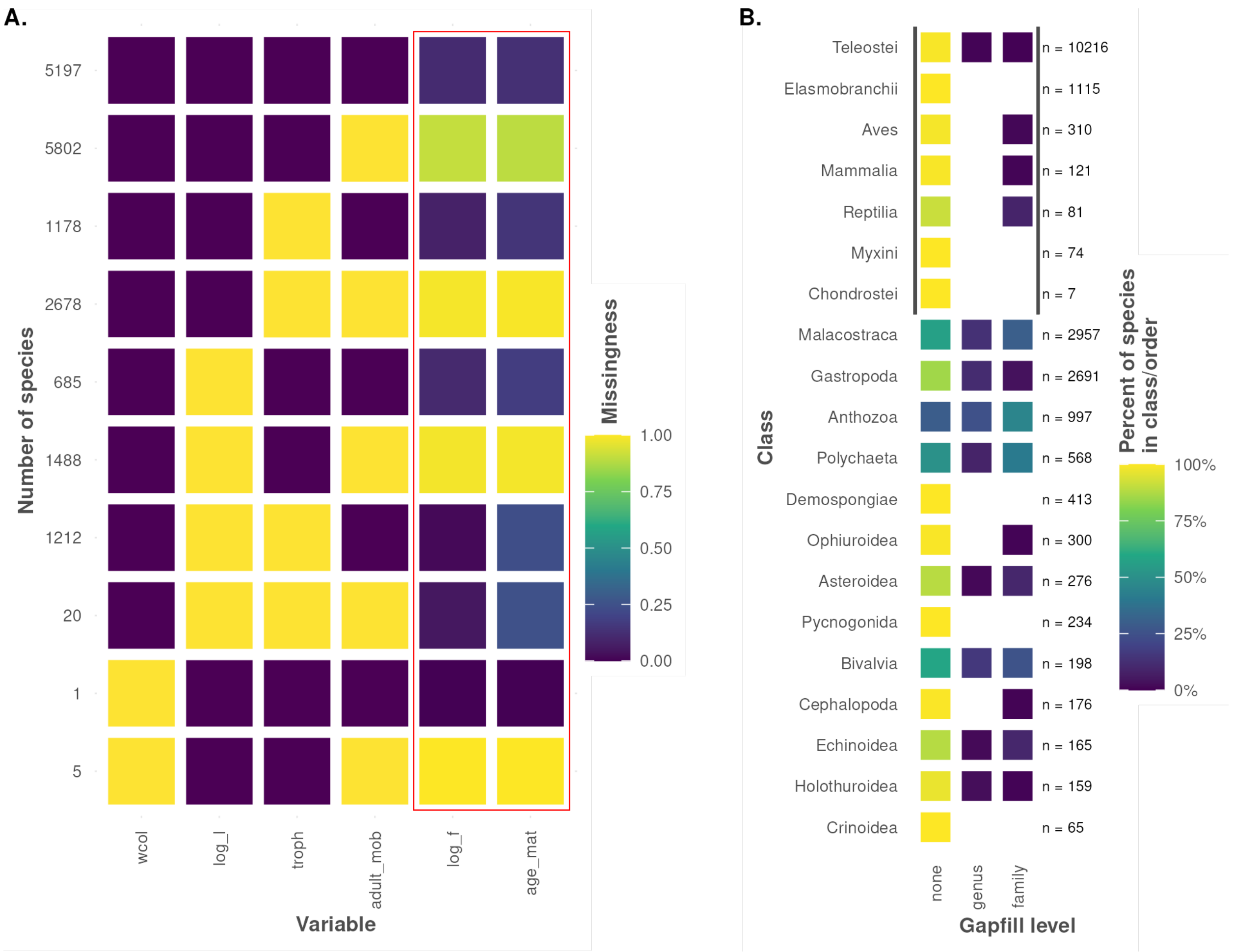

Figure S7. Trait imputation. A) Missingness of traits for inclusion in functional entities, prior to MICE imputation. Water column position (`wcol`), log(body length) (`log_l`), trophic level (`troph`), and adult mobility (`adult_mob`) were used to assign species to functional entities; dark boxes represent known values, yellow boxes represent missing values. Two other traits highlighted in the red box, log(fecundity) (`log_f`) and age to maturity (`age_mat`) were used to assist in imputation where available; color scale represents proportional missingness of those traits for each combination of the other four. B) After MICE imputation, an additional gapfill step using taxonomic relatives was used. "None" represents proportion of species successfully imputed by MICE; "genus" represents proportion of species whose traits were gapfilled using the most common values of other species in its genus; and "family" represents the proportion filled using the most common values of other species in its family.

Supporting Tables

Table S1: Species inclusion by phylum and class. N total refers to the number of marine species in each class according to the World Register of Marine Species [28], N included refers to the number of species included in this assessment.

Phylum	Class	N included	Subpops/ synonyms	N total	% included
Annelida	Polychaeta	568	-	10449	5.4%
Arthropoda	Malacostraca	2957	2	33502	8.8%
Arthropoda	Pycnogonida	234	-	1366	17.1%
Chordata	Teleostei	10216	1	18538	55.1%
Chordata	Elasmobranchii	1115	-	1249	89.3%
Chordata	Aves	310	2	341	90.9%
Chordata	Mammalia	121	-	224	54%
Chordata	Reptilia	81	-	105	77.1%
Chordata	Myxini	74	2	87	85.1%
Chordata	Chondrostei	7	-	22	31.8%
Cnidaria	Anthozoa	997	2	9403	10.6%
Echinodermata	Ophiuroidea	300	-	2330	12.9%
Echinodermata	Asteroidea	276	-	2195	12.6%
Echinodermata	Echinoidea	165	-	3317	5%
Echinodermata	Holothuroidea	159	-	1790	8.9%
Echinodermata	Crinoidea	65	-	667	9.7%
Mollusca	Gastropoda	2691	27	37523	7.2%
Mollusca	Bivalvia	198	-	10992	1.8%
Mollusca	Cephalopoda	176	-	838	21%
Porifera	Demospongiae	413	-	7482	5.5%

Table S2: Vertebrate species inclusion by class and order. N total refers to the number of marine species in each order according to the World Register of Marine Species [28], N included refers to the number of species included in this assessment.

Class	Order	N included	Subpops/ synonyms	N total	% included
Teleostei	Perciformes	1573	-	3237	48.6%
Teleostei	Eupercaria incertae sedis	1344	-	1765	76.1%
Teleostei	Blenniiformes	910	-	957	95.1%
Teleostei	Gobiiformes	625	-	1806	34.6%
Teleostei	Anguilliformes	442	-	1019	43.4%
Teleostei	Pleuronectiformes	436	-	794	54.9%
Teleostei	Ovalentaria incertae sedis	410	-	789	52%
Teleostei	Acanthuriformes	380	-	447	85%
Teleostei	Tetraodontiformes	337	-	420	80.2%
Teleostei	Gadiformes	324	-	647	50.1%
Teleostei	Clupeiformes	301	1	352	85.5%
Teleostei	Syngnathiformes	279	-	315	88.6%
Teleostei	Stomiiformes	249	-	443	56.2%
Teleostei	Kurtiformes	229	-	387	59.2%
Teleostei	Myctophiformes	216	-	272	79.4%
Teleostei	Ophidiiformes	210	-	554	37.9%
Teleostei	Lophiiformes	191	-	397	48.1%
Teleostei	Scombriformes	182	-	267	68.2%
Teleostei	Aulopiformes	175	-	296	59.1%
Teleostei	Carangiformes	161	-	175	92%
Teleostei	Beloniformes	130	-	204	63.7%
Teleostei	Acropomatiformes	120	-	288	41.7%
Teleostei	Centrarchiformes	107	-	189	56.6%
Teleostei	Callionymiformes	83	-	217	38.2%
Teleostei	Alepocephaliformes	69	-	142	48.6%
Teleostei	Holocentriformes	61	-	91	67%
Teleostei	Carangaria incertae sedis	59	-	95	62.1%
Teleostei	Mulliformes	58	-	100	58%
Teleostei	Siluriformes	56	-	259	21.6%
Teleostei	Beryciformes	55	-	124	44.4%
Teleostei	Gobiesociformes	53	-	178	29.8%
Teleostei	Argentiniformes	52	-	97	53.6%
Teleostei	Other teleostei	339	-	1215	27.9%
Elasmobranchii	Carcharhiniformes	279	-	300	93%
Elasmobranchii	Rajiformes	279	-	320	87.2%
Elasmobranchii	Myliobatiformes	181	-	219	82.6%
Elasmobranchii	Squaliformes	136	-	142	95.8%
Elasmobranchii	Rhinopristiformes	72	-	85	84.7%
Elasmobranchii	Torpediniformes	61	-	70	87.1%
Elasmobranchii	Orectolobiformes	45	-	45	100%
Elasmobranchii	Squatiniformes	21	-	25	84%
Elasmobranchii	Lamniformes	15	-	16	93.8%
Elasmobranchii	Heterodontiformes	9	-	9	100%
Elasmobranchii	Pristiophoriformes	9	-	10	90%
Elasmobranchii	Hexanchiformes	6	-	6	100%
Elasmobranchii	Echinorhiniformes	2	-	2	100%
Aves	Charadriiformes	115	-	116	99.1%
Aves	Procellariiformes	115	2	130	88.5%
Aves	Pelecaniformes	35	-	50	70%
Aves	Anseriformes	18	-	18	100%
Aves	Sphenisciformes	18	-	18	100%
Aves	Gaviiformes	5	-	5	100%
Aves	Podicipediformes	4	-	4	100%
Mammalia	Cetartiodactyla	84	-	89	94.4%
Mammalia	Carnivora	34	-	70	48.6%
Mammalia	Sirenia	3	-	63	4.8%
Reptilia	Squamata	71	-	93	76.3%
Reptilia	Testudines	7	-	8	87.5%
Reptilia	Crocodilia	2	-	3	66.7%
Reptilia	Sauria	1	-	1	100%
Myxini	Myxiniformes	74	2	87	85.1%
Chondrostei	Acipenseriformes	7	-	22	31.8%

Table S3: Traits used to calculate vulnerability to various stressors, according to sensitivity (S), specific adaptive capacity (A), general adaptive capacity (G), and binary exposure modifier (0/1) components. Where marked, different values of that trait change the value of that component. See [19] for full details and methodology. Note that sensitivity to Biomass Removal and SST Rise stressors (noted with *) are set to 1 for all species, but vulnerability to these stressors is moderated by adaptive capacity and exposure modifier.

	Category	Trait	Bycatch	Nutrient pollution	Habitat loss	Light pollution	Ocean acidification	Sea level rise	Ultraviolet	SST extremes	Wildlife strike	Biomass removal*	SST rise*
Sensitivity	Reproductive Traits	feeding larva (post-hatching metamorphosis)		S		S	S		S	S			
	Physiological tolerance	thermal tolerance range								S			
		thermal - sensitivity to heat spikes/heat waves								S			
		salinity tolerance		S									
		pH range		S			S						
		dissolved oxygen range		S						S			
		sensitivity to wave energy (physical forcing)		S									
	Specialisation	photosynthetic		S				S	S				
		air-sea interface	S	S						S	S		
		within-stage dependent habitats + condition			S								
		across-stage dependent habitats + condition			S								
		habitat forming			S								
		terrestrial and marine life stages			S	S		S		S			
		extreme diet specialization		S	S					S			
		dependent interspecific interactions			S					S			
	Biophysical Traits	adult body mass/body size	S								S		
		calcium carbonate structure location		S			S			S			
		calcium carbonate structure life stages		S			S			S			
		biomineral		S			S			S			
		navigation requirements (light)		S		S							
		respiration structures		S							S		
Specific Adaptive Capacity	Movement	adult mobility		A	A	A	A	A	A	A	A		
		planktonic larval duration (PLD) exposure		A			A		A				
General Adaptive Capacity	Spatial Scale of species	EOO (range)	G	G	G	G	G	G	G	G	G	G	G
	Reproductive Traits	reproductive strategy	G	G	G	G	G	G	G	G	G	G	G
		fecundity	G	G	G	G	G	G	G	G	G	G	G
		lifetime # reproductive opportunities	G	G	G	G	G	G	G	G	G	G	G
		age to 1st reproduction/generation time	G	G	G	G	G	G	G	G	G	G	G
		max age	G	G	G	G	G	G	G	G	G	G	G
		parental investment	G	G	G	G	G	G	G	G	G	G	G
		post-birth/hatching parental dependence	G	G	G	G	G	G	G	G	G	G	G
		global population size	G	G	G	G	G	G	G	G	G	G	G
		are there sub-populations?	G	G	G	G	G	G	G	G	G	G	G
	Breeding/nesting/spawning aggregations	number of sites	G	G	G	G	G	G	G	G	G	G	G
		sub-population dependence on particular sites	G	G	G	G	G	G	G	G	G	G	G
	foraging range	number of sites	G	G	G	G	G	G	G	G	G	G	G
		sub-population dependence on particular sites	G	G	G	G	G	G	G	G	G	G	G
Exposure Modifier	Spatial Scale of species: depth	air	0	0	1	1	0	1	1	0	1	1	1
		epipelagic	1	1	1	1	1	1	1	1	1	1	1
		mesopelagic	1	0	1	0	1	0	0	0	0	1	0
		bathypelagic	1	0	1	0	1	0	0	0	0	1	0
		abyssopelagic	0	0	0	0	1	0	0	0	0	1	0
		hadopelagic	0	0	0	0	1	0	0	0	0	1	0
	Spatial Scale of species: Zone	intertidal	1	1	0	1	1	1	1	1	0	1	1
		neritic	1	1	1	1	1	1	1	1	1	1	1
		oceanic	1	0	1	0	1	0	0	0	0	1	0
		demersal	1	1	1	0	1	0	0	1	0	1	0
		benthic	1	1	1	0	1	0	0	1	0	1	0

Table S4: Overview of methods and data sources to generate stressor distribution maps. All stressors projected to 10 km Mollweide coordinate reference system.

Stressor	Category	Exposure	Preparing data	Transform	Rescaling (0 to 1)	Potential species-level impacts	Data citation
Sea surface temperature extremes	Climate	Uniform across all species or habitat	Number of extreme SST weeks during a five-year period (2016-2020) . An extreme week was defined as a weekly anomaly (weekly SST - weekly climatological SST) exceeding the 90th quantile of anomalies calculated across 1985-2015 for that week.	NA	Number of extreme weeks divided by the total possible extreme events	Physiological stress or mortality	[75]
Sea surface temperature rise	Climate	Per species	Average SST from 2016-2020.	NA	Rescaled relative to each species's historical thermal tolerance.	Physiological stress or mortality	[75]
Ocean acidification	Climate	Uniform: species, habitat	Monthly aragonite saturation values (Ω) averaged to obtain annual estimate for 2017.	NA	Cell stressor score = 1 when Ω ≤ 1; otherwise, stressor score = (Ω_current - Ω_ref) / (1 - Ω_ref) where Ω_ref = average from 1880-1889	Disruption of calcium structure formation, physiological stress or mortality	[76]
Ultraviolet radiation	Climate	Uniform: species, habitat	Number of extreme weekly events occurring from 2016-2020 minus the extreme events from a reference period from 2005-2009. An extreme event is defined as a week that exceeds the mean UV + 1sd calculated for each week from 2004-2016.	NA	Normalized by raster value corresponding 99.9th quantile	Planktonic larval mortality	[77]
Sea level rise	Climate	Uniform: species, habitat	Monthly anomalies averaged to obtain annual mean sea level anomaly. 5 year mean of annual data, 2015 to 2019 used to smooth large yearly variation.	NA	Normalized by raster value corresponding 99.9th quantile	Flooding, habitat disruption	[78]
Nutrient pollution (runoff)	Non-climate (land-based)	Uniform: species, habitat	Intensity (tonnes/km2) of pollution from 2017 values of land-based nitrogen effluent from synthetic fertilizer and manure, human wastewater, and aquaculture.	NA	Normalized by raster value corresponding 99.9th quantile	Eutrophication, harmful algal blooms, physiological stress or mortality	[7,79,80]
Direct human disturbance	Non-climate (land-based)	Uniform: species, habitat	Total human population living within 25 km of the shoreline for 2020 For each raster cell, coastal human population summed for a 25 km radius. Data cropped to include only cells 3nm from the coast.	ln(x') where x' = x + max(x)/100	Normalized by raster value corresponding 99.9th quantile	Intertidal habitat destr., coastal development, habitat fragmentation	[81]
Light pollution	Non-climate (land-based)	Uniform: species, habitat	Harmonized DMSP/VIIRS nighttime light data, using minimum value between 2017 and 2018 layers and dropping values below 10 (of 63) to reduce artifacts.	NA	Normalized by raster value corresponding 99.9th quantile (excluding zero values)	Disruption of navigation	[82]
Shipping	Non-climate (ocean-based)	Uniform: species, habitat	Ship tracks in 2020 from AIS transponders at 0.005 degree resolution. Summed commercial, passenger, and oil/gas ship tracks and transformed to 10 km Mollweide	none	Normalized by raster value corresponding 99.9th quantile	Ship strikes, noise pollution, chemical pollution	[83]

Stressor	Category	Exposure	Preparing data	Transform	Rescaling (0 to 1)	Potential species-level impacts	Data citation
			analysis grid.				
Benthic structures	Non-climate (ocean-based)	Uniform: species, habitat	Oil/gas rigs in 2020 from AIS transponders at 0.005 degree resolution with at least 5 pings in the same position. Global offshore wind turbines identified from Sentinel-1 SAR. Modeled rig benthic footprint.	ln(x')	Normalized by maximum observed value	Benthic habitat disruption or conversion	[83,84]
Biomass removal (targeted fishing)	Non-climate (fishing)	Per species	Total tonnes of catch from industrial and nonindustrial fisheries calculated for 2015-2017 (0.5° resolution). Catch divided by Net Primary Productivity to standardize by region's productivity.	NPP ln(x'), catch not transformed	Reference point per species	Biomass removal	[33,85–87]
Habitat destr: demersal destructive fishing	Non-climate (fishing)	Uniform: species, habitat	Hours of fishing effort per raster cell using destructive fishing gear in 2017.	ln(x')	Normalized by raster value corresponding 99.9th quantile	Benthic habitat disruption or conversion	[88,89]
Bycatch (pelagic)	Non-climate (fishing)	Species position in water column	Total tonnes of discards for industrial and non-industrial fishing from 2015-2017. Pelagic bycatch based on non-demersal to standardize by local productivity.	NPP ln(x'), catch not transformed	Normalized by raster value corresponding 99.9th quantile	Biomass removal	[33,85–87]
Bycatch (benthic)	Non-climate (fishing)	Species position in water column	Total tonnes of discards for industrial and non-industrial fishing from 2015-2017. Benthic bycatch based on demersal gear types and normalized by bottom NPP, sum of mean bottom NPP and exponential decay of surface NPP to estimate benthic nutrients from marine snow.	NPP ln(x'), catch not transformed	Normalized by raster value corresponding 99.9th quantile	Biomass removal	[33,85–87]
Fishing: Artisanal	Non-climate (fishing)	Habitat	Total tonnes of catch from nonindustrial fisheries calculated for 2015-2017. Catch divided by Net Primary Productivity to standardize by region's productivity.	NPP ln(x'), catch not transformed	Normalized by raster value corresponding 99.9th quantile	Biomass removal	[33,85–87]
Fishing: demersal destructive	Non-climate (fishing)	Habitat	Total tonnes of catch for industrial demersal fishing using gear types causing habitat destruction calculated for 2015-2017. Catch divided by Net Primary Productivity to standardize by region's productivity.	NPP ln(x'), catch not transformed	Normalized by raster value corresponding 99.9th quantile	Biomass removal	[33,85–87]
Fishing: demersal nondestructive high bycatch	Non-climate (fishing)	Habitat	Total tonnes of catch for industrial demersal fishing using high bycatch practices calculated for 2015-2017. Catch data divided by Net Primary Productivity to standardize by the region's productivity.	NPP ln(x'), catch not transformed	Normalized by raster value corresponding 99.9th quantile	Biomass removal	[33,85–87]
Fishing: demersal nondestructive low bycatch	Non-climate (fishing)	Habitat	Total tonnes of catch for industrial demersal fishing using low bycatch practices calculated for 2015-2017. Catch data divided by Net Primary Productivity to standardize by the region's productivity.	NPP ln(x'), catch not transformed	Normalized by raster value corresponding 99.9th quantile	Biomass removal	[33,85–87]
Fishing: pelagic high bycatch	Non-climate (fishing)	Habitat	Total tonnes of catch for industrial pelagic fishing using high bycatch practices calculated for 2015-2017. Catch data divided by Net Primary Productivity to standardize by region's productivity.	NPP ln(x'), catch not transformed	Normalized by raster value corresponding 99.9th quantile	Biomass removal	[33,85–87]
Fishing: pelagic low bycatch	Non-climate (fishing)	Habitat	Total tonnes of catch for industrial pelagic fishing using low bycatch practices calculated for 2015-2017. Catch data divided by Net Primary Productivity to standardize by region's productivity.	NPP ln(x'), catch not transformed	Normalized by raster value corresponding 99.9th quantile	Biomass removal	[33,85–87]

Supporting Methods

Analysis grid

All spatial analyses were calculated on a gridded global map using a Mollweide equal-area projection coordinate reference system (CRS), gridded to 10 km x 10 km resolution. An ocean base map was prepared by rasterizing the vector ocean polygon features of the Natural Earth (https://www.naturalearthdata.com/) Oceans 1:10m dataset to a 1 km x 1 km Mollweide projection, then aggregating by a factor of 10 to approximate percentage of ocean within each cell. The resulting 10 km Mollweide ocean raster was used as the target raster for projecting all other datasets, and was used to mask out non-ocean cells from reprojected data.

To examine variation in calculations across coastal regions and ecoregions, we prepared two supporting rasters at the same CRS as our analysis grid: coastal area based on cells with a minimum depth less than 200 m, based on GEBCO bathymetry data [90], and marine ecoregions of the world [46].

Species distributions

Species distribution data were taken from AquaMaps [30] (n = 18,480) and IUCN species distribution maps [31,32] (n = 2,679). For both datasets, synonymous scientific binomials and slight differences in nomenclature were resolved by comparing names against accepted names of marine species in the World Register of Marine Species (WoRMS, [29]) using the `taxize` package [43,44]. For species appearing in both distribution map datasets, the AquaMaps distribution maps, based on transparent and repeatable algorithms using publicly available data, were preferred over IUCN range maps, which integrate data and expert knowledge but may include mapping decisions that are difficult to replicate. Each mapping method has advantages and disadvantages, though neither method can perfectly replicate the underlying truth [91].

AquaMaps predicts species ranges as "probability of occurrence" in 0.5° cells, computed based on long-term averages of species-specific relative environmental suitability across multiple environmental variables (temperature, salinity, depth, sea ice concentration, primary production, and in some cases oxygen level and distance to shore) based on species-specific habitat preference derived from publicly available occurrence data [30]. To determine the range map for each species, we converted gradient probabilities into binary presence/absence using a threshold of ≥0.5 to represent "presence" of a given species, following a common practice in conservation literature [e.g., 50,92–94]. Species maps based on fewer than 10 "occurrence cells" (0.5° cells with at least one validated occurence record) were rejected to ensure quality [30]. These results were then reprojected in raster form to the Mollweide CRS at 10 km resolution, resulting in n = 18,480 species presence maps. AquaMaps outputs have been validated against independent survey occurrence data and other algorithms often used in species distribution modeling [95,96].

The IUCN dataset presents species ranges as polygons representing the historical, present and possible distribution of a taxon's occurrences [31]. For each species (or subpopulation where available), we excluded polygons with a "presence" value of 5 indicating "extinct" portions of a

range, reprojected the remaining polygon features to the Mollweide CRS, then rasterized the results to the 10 km analysis grid, resulting in n = 2,679 species presence maps.

For coastal and neritic species from both datasets, we masked the resulting presence maps to cells with a minimum depth of 200 m or less using GEBCO bathymetry data [90], and masked using the ocean area raster to exclude non-ocean cells. The depth mask for shallow-water species is an effective method of reducing errors of commission in IUCN range maps to better align them with AquaMaps distribution maps [97]. While AquaMaps uses bottom depth as a parameter to limit ranges for depth-limited species, this masking method allows for a finer resolution map than the 0.5 degree native resolution of AquaMaps.

Vulnerability estimates

Vulnerability weights, i.e., relative effect of stressor j on the fitness/health of species i, were determined based on methods of Butt et al. [19]. That study estimated vulnerability of species i to stressor j based on presence of certain traits that are likely to increase the species' physiological sensitivity to the stressor S_{ij}, other traits that affect the species' ability to adapt to that specific stressor, i.e., stressor-specific adaptive capacity A_{ij}, and life history and population-level traits that affect the population's ability to adapt to or recover from disturbances in general, i.e., general adaptive capacity G_i. Traits were binned into nominal or ordinal categories and certain trait values corresponded to different scores for different stressors (see S3 Table). An additional exposure modifier was included to account for possibility of exposure $E_{ij} \in \{0,1\}$ of species i to stressor j, e.g., a mesopelagic species (depth below 200 m) will not be exposed to ship strikes, so $E_{ij} = 0$. These metrics were combined to produce a vulnerability score $V_{ij} \in [0,1]$:

$$V_{ij} = \frac{S_{ij}/S_{ij,max}}{1 + A_{ij}/A_{ij,max} + G_i/G_{i,max}} \times E_{ij}$$

For the present study, we updated some aspects of methodology to improve imputation of species vulnerability for species with partial trait sets. First, for species whose traits were provided by taxon experts at genus level or higher rank in the trait set assembled by Butt et al. [19], certain traits (i.e., body length, fecundity, generation time, temperature tolerances, and depth preferences) were filled using species-level data (where available) from FishBase/SealifeBase [36,37] and AquaMaps [30], while extent of occurrence was determined from the species' distribution map as described above. Remaining traits were taken as provided by the taxon experts for the original study. Second, rather than calculate sensitivity, adaptive capacity, and vulnerability scores for species groups then impute missing values based on the distribution of those scores (as done in the original study), we first imputed missing trait values based on frequency within the species' taxonomic neighbors, then scored the sensitivity, adaptive capacity, and vulnerability from the combination of species-level traits (from FishBase, SeaLifeBase, and AquaMaps) and those imputed traits. Note that these methodological updates resulted in small differences in the vulnerability scores between the published study and our present study: mean/sd difference of .0002 $\pm$ .005, with 98% of differences between -0.00009 and 0.00024.

The expert-elicited trait values used to calculate vulnerability were given as ordinal or nominal categorical values, generally with a single value attributed to each trait for species. While a single value does not account for large-scale interspecific variability or regional variations in traits, most traits are broadly applicable across a species, genus, or family (e.g., respiration structures of gills vs. lungs vs. diffusion), and for traits representing continuous values (e.g., body size, fecundity, age to maturity), the bins typically increase as orders of magnitude, wide enough to accommodate substantial variation without affecting the vulnerability estimate.

The traits used to calculate sensitivity, adaptive capacity, and exposure components of vulnerability for each stressor are included in S3 Table. The updated trait-based vulnerability methods and results, including the full matrix translating trait values to vulnerability components and the full dataset of species traits, can be found at https://github.com/mapping-marine-spp-vuln/spp_vuln_framework.

Uniform exposure stressors

For most of the included stressors, exposure does not depend on species identity (though vulnerability to the stressor certainly might) and therefore we modeled exposure as uniform across all species. For these stressors, a single map of relative stressor intensity was created from gridded data using the following general process:

- We reprojected raw intensity to Mollweide CRS at 10 km resolution
- For stressors where marginal impact is expected to be decreasing with intensity (e.g., the hundredth hour of trawling in an area likely overlaps habitat already destroyed by the first hour of trawling), we applied a log transformation to the raw data.
- For stressors whose distribution contains a small number of extreme outliers, we identified a reference point based on the 99.9th percentile; otherwise we assigned a reference point based on the maximum observed value.
- Finally, we rescaled the data using the reference point to result in a distribution of stressor intensity ranging between zero and one.

Information on the data source, transformation, and reference point used for each stressor layer can be found in S4 Table.

Non-uniform exposure stressors

For several stressors, exposure intensity depends on species-specific information. These stressors include bycatch, targeted fishing, and sea surface temperature rise.

Bycatch stressor

The degree to which species are exposed to bycatch is dependent on their position in the water column. Pelagic species are unlikely to be swept up in a bottom trawl, while demersal species are unlikely to be swept up in a midwater trawl or purse seine. We prepared three bycatch layers,

summing industrial and nonindustrial discards based on gear type listed by Watson [33] and Watson et al. [85]:

- Benthic bycatch (affects species identified as benthic), based on gear types: trawl, dredge, and trap
- Pelagic bycatch (affects species identified as pelagic), based on gear types: line (tuna and non-tuna), longline (tuna and non-tuna), midwater trawl, seine, purse seine (tuna and non-tuna), gillnet, other
- Both (affects species identified as benthopelagic or reef-associated): the average of benthic and pelagic bycatch layers.

Catch estimates in Watson [33] and Watson et al. [85] provide data on discards (industrial/non-industrial) by gear type, presented in 0.5° cells. The discard values were summed across benthic or pelagic gear types, then the totals were normalized by cell ocean area resulting in an intensity of discarded catch, i.e., tonnes of bycatch per square kilometer. These intensity rasters were reprojected to the 10 km Mollweide CRS analysis grid. The intensity rasters were then adjusted by dividing by ln(NPP) according to water column position, to indicate that bycatch in a high productivity area is less problematic than the same amount of bycatch in a low productivity area.

Surface NPP data were taken from Bio-ORACLE [86,87], mean sea surface net primary productivity (NPP) of carbon, g/m^3/day.

Benthic NPP sums productivity at bottom depth and export flux (e.g., "marine snow") from the surface to bottom depth. Bottom NPP data were taken from Bio-ORACLE [86,87], mean NPP of carbon at mean bottom depth, g/m^3/day. Export flux from surface to bottom depth were calculated based on an exponential decay model for export flux at depth d:

$$f(d) = f_0 \times (1 - r)^{d^\theta}$$

Applying non-linear least squares using data from Table 1 (control) in Gt C a^{-1} (globally integrated) in Yool et al [98], we identified best fit parameters $r = 0.341, \theta = .288$.

Finally, the resulting surface and benthic catch/ln(NPP) layers were rescaled from 0 to 1, using a reference point based on the 99.9% quantile of observed cell values.

Targeted fishing stressor

In addition to discards, Watson [33] and Watson et al. [85] report targeted catch for industrial and non-industrial fisheries at 0.5° cells, across multiple gear types and taxonomic groups. While any species might be vulnerable to targeted fishing, not all species are targeted, and so the targeted fishing stressor layer is distinct for every targeted species (and nonexistent for non-targeted species), thus a targeted fishing stressor layer was calculated separately for each species with non-zero catch in the Watson dataset.

Taxon names were compared to accepted names per WoRMS [29] using the `taxize` package [43,44] to resolve synonyms and differences in spelling. Total catch for each taxon was summed across pelagic gears and benthic gears separately, then divided by cell ocean area resulting in intensity, i.e., tonnes of catch per km^2. Catch reported at the species level was attributed directly to that species. Catch in a given cell but reported at higher ranks (e.g., genus, family) was divided

equally among all local species (per species distributions) in that genus or family. In many cases, a given species would be attributed catch at multiple taxonomic levels in the same cell, which were summed to create a cell total catch intensity for each species, separately for pelagic and benthic gear types. The pelagic and benthic catch intensities were reprojected to the 10 km Mollweide CRS analysis grid, and then normalized by ln(NPP), either surface or benthic as appropriate, to account for the fact that a unit of catch in a highly productive region imposes less stress on an ecosystem than the same unit of catch in a low-productivity region. The pelagic and benthic NPP-normalized catch were then summed for each cell for the species.

Reference points to rescale the targeted fishing stressor layers are species specific. A global maximum reference point was set by first calculating the 90th percentile of NPP-normalized catch for each species across its entire distribution, then selecting the score of the species with the highest 90th percentile value: *Engraulis ringens*, Peruvian anchoveta, at C_{ref} = 2,170 tonnes of NPP-normalized catch. This global reference point was used to rescale (from 0 to 1) any species whose 99.9th percentile of NPP-normalized catch exceeded this value (15 species total). For species whose 99.9th percentile catch across its range falls below this reference point, we used the 99.9th percentile of that species' catch across its range as its own reference point. The reference catch for species i is therefore:

$$C^i_{ref} = \min\left(C_{ref}, C^i_{99.9\%}\right)$$

The NPP-normalized catch was then rescaled using the appropriate reference point, with values capped at 1.0, resulting in a gridded map of stressor values from 0 to 1 for every species with non-zero targeted catch.

SST rise stressor

We included two stressors related to ocean temperature: sea surface temperature extremes, representing impacts from short-lived (weeks to months) high temperature events, i.e., marine heat waves; and rise in annual mean sea surface temperature representing long-term (years to decades) changes in sea surface temperature relative to a historic baseline. The SST extremes stressor is described above in the uniform-exposure stressors. Exposure to long-term SST rise estimates the impact to a species when mean annual temperatures risk exceeding the physiological tolerance of the species due to the climatic shift from historic norms in a given location. For species included in the AquaMaps dataset, we used the thermal preference envelope used to generate species distributions; for species included in IUCN but not AquaMaps, we generated thermal preference envelopes (absolute and preferred minimum and maximum temperatures) in a manner similar to that used to generate envelopes for AquaMaps, using observed mean annual temperature in cells across the species distribution according to IUCN distribution maps:

- T^a_{min} = (25th percentile - 1.5 × interquartile) or absolute minimum mean annual temperature (whichever is lesser)
- T^a_{max} = 75th percentile + 1.5 × interquartile or absolute maximum mean annual temperature (whichever is greater)
- T^p_{min} = 10th percentile of observed variation in mean annual temperature

- T^p_{max} = 90th percentile of observed variation in mean annual temperature

We modeled physiological thermal stressor intensity s_T for each species based on the local mean annual temperature $\bar{T}$ relative to its preferred and absolute thermal range:

$$s_T = \begin{cases} 0 & \text{where } \bar{T} \leq T^p_{max} \\ \dfrac{\bar{T} - T^p_{max}}{T^a_{max} - T^p_{max}} & \text{where } T^p_{max} < \bar{T} < T^a_{max} \\ 1 & \text{where } \bar{T} \geq T^p_{max} \end{cases}$$

For each species, mean annual temperature from [75] in each pixel across its distribution was compared to the thermal preferences according to the above formula to generate a species-specific map of thermal stressor intensity. Note that species whose minimum depth preference was deeper than 200 meters (i.e., not epipelagic) were assigned a value of zero for this stressor.

Stressor layers for habitat method

Stressor layers for the Habitat Method of calculating cumulative human impact included all the same uniform exposure stressor layers used for the species and functional entity methods. See S4 Table for details on transformations and rescaling.

Species-specific stressor layers (SST rise stressor based on species thermal tolerance, targeted fishing based on species identity, benthic and pelagic bycatch based on position in the water column) could not be included in the same manner as for the species approach, as those calculations depended on species-specific information that cannot be broadly applied at the representative habitat level. However, fisheries pressures (targeted and bycatch) were accounted for by creating layers from the same source, i.e., Watson [33], in the same manner as described in Halpern et al. [7]. These new layers were aggregated by method, depth, and scale: commercial pelagic and demersal low bycatch, commercial pelagic high bycatch, commercial demersal destructive, and artisanal/small scale fishing. For all these categories, overall fishing intensity (catch per km^2) was normalized by ln(NPP), then rescaled to the 99.9% quantile across all years for that catch category, resulting in stressor scores from 0 to 1. See S4 Table for details on transformations and rescaling.

Habitat maps for habitat method

Habitat maps prepared for Halpern et al. [7], at 934 m resolution in a Mollweide equal area coordinate reference system, were aggregated by a factor of 11 (to identify habitat density at approximately 10 km resolution) then reprojected to match our analysis grid. Corals and seagrass layers were based on habitat maps updated for Berger et al. [79]. Kelp and saltmarsh layers were based on maps updated for the Ocean Health Index 2021.

Functional entities

We assigned species to functional entities based on categorical values of four traits (maximum body length, adult mobility, water column position, adult trophic level) that roughly gather species into similar niche space, following Mouillot et al. [26]. Out of 512 possible functional entities (8 body

length categories × 4 adult mobility categories × 4 water column position × 4 trophic level categories = 512 unique combinations of trait values), 339 contained at least one species within our set (min: 1 species; max: 1034 species; mean: 63.59; median: 19). Due to limited trait data available across a broad range of taxa, our analysis relied on a smaller set of traits for assignment of functional entity than Mouillot et al. [26], which results in fewer but more populous functional entities and therefore a more conservative estimate of functional vulnerability.

Trait values were gleaned from [19,36,37]; missing values were imputed using Multiple Imputation by Chained Equation (MICE) in the R package `mice` [38] using all other traits plus fecundity (where available), generation time (where available), order, and family. S7 Fig. shows missingness of traits that were gapfilled.

Maximum body length

Values of maximum total length (in cm) were determined from sources: [19,36,37]

- (0, 1.5]
- (1.5, 7.0]
- (7, 15]
- (15, 30]
- (30, 50]
- (50, 80]
- (80, 150]
- (150, …)

For categorizing maximum body length, we largely relied on the same values as Mouillot et al. [26], adding a bin on either end to capture the wider range of values possible in our set of species.

Adult mobility

Values were determined from sources: [19]

- Sessile
- Sedentary
- Mobile resident
- Migratory/nomadic

Water column position

Values were determined from sources: [19,36,37]

- Benthic
- Benthopelagic
- Pelagic
- Reef-associated

Adult trophic level

Values were determined from sources: [36,37]

- Primary consumer: (1, 2]
- Secondary consumer: (2, 3]
- Tertiary consumer: (3, 4]
- Apex consumer: (4, …)

Sensitivity analysis: trait selection

To test the sensitivity of the functional entity approach to variation in assignment of trait values, we chose a random sample of 100,000 cells and subjected the species compositions in each cell to a Monte Carlo simulation of trait values. For each trait used to identify functional entity membership (maximum body length, adult trophic level, adult mobility, and water column position), trait values within each cell were resampled with replacement (holding the other three traits constant) and reassigned to the species within the cell. Based on the reassigned trait values, species were regrouped into new functional entities, and the functional vulnerability calculation was performed on this new set. The resample procedure was performed 1000 times for each trait, with a unique pseudorandom seed assigned to each simulation to ensure reproducibility.

From the 1000 simulations for each trait, summary statistics (mean, std deviation, 95% confidence interval) were calculated for functional vulnerability. The difference between the simulated mean and the base calculation is shown in S6 Fig. A-D. The randomly resampling and reassigning of trait values results in novel trait combinations not likely to be found in nature, resulting in more low-membership functional entities, and thus increasing the average functional vulnerability across the simulated community (mean 0.037, std dev 0.040).

To measure the effect of trait variation on cumulative impact, we calculated the cumulative impact according to the functional entity method on each simulated community. Note that while some of these traits were used to estimate a species' vulnerability to a stressor, we did not recalculate the species-stressor vulnerability score here, only the functional entity membership. The difference between the simulated mean cumulative impact and the base calculation is shown in S6 Fig. E-H. Even considering the slightly elevated functional vulnerability, the difference in cumulative impact was quite small (mean -0.0026, std dev 0.019).

R code and packages

All analysis was performed in R statistical software, version 4.0.4 [39], relying primarily on packages `tidyverse` [40], `terra` [41], `sf` [42], `taxize` [43,44], `rfishbase` [45].

All data used as inputs for this assessment are freely available from original sources. All original code and data resulting from this analysis has been deposited at Figshare and is publicly available as of the date of publication. DOI: 10.6084/m9.figshare.26454106. A living version of the code and data can be found at https://github.com/mapping-marine-spp-vuln/spp_vuln_mapping.

Supporting References

75. Saha K, Zhao X, Zhang H, Casey KS, Zhang D, Zhang Y, et al. The Coral Reef Temperature Anomaly Database (CoRTAD) Version 6 - Global, 4 km Sea Surface Temperature and Related Thermal Stress Metrics for 1982 to 2020. [Weekly SST and SSTA]. NOAA National Centers for Environmental Information. Dataset; 2020.

76. Feely RA, Doney SC, Cooley SR. Ocean Acidification: Present Conditions and Future Changes in a High-CO_2 World. Oceanography. 2009;22: 36–47. Available: https://www.jstor.org/stable/24861022

77. Hovila J, Arola A, Tamminen J. UVB Irradiance and Erythemal Dose Daily L2 Global Gridded 0.25 degree x 0.25 degree V3. NASA Goddard Space Flight Center, Goddard Earth Sciences Data and Information Services Center (GES DISC); 2014.

78. Ssalto/Duacs. AVISO MSLA heights, monthly means. Aviso+; 2021.

79. Berger M, Canty SWJ, Tuholske C, Halpern BS. Sources and discharge of nitrogen pollution from agriculture and wastewater in the Mesoamerican Reef region. Ocean & Coastal Management. 2022;227: 106269. doi:10.1016/j.ocecoaman.2022.106269

80. Clawson G, Kuempel CD, Frazier M, Blasco G, Cottrell RS, Froehlich HE, et al. Mapping the spatial distribution of global mariculture production. Aquaculture. 2022;553: 738066. doi:10.1016/j.aquaculture.2022.738066

81. Center for International Earth Science Information Network - CIESIN - Columbia University. Gridded Population of the World, Version 4 (GPWv4): Population Density Adjusted to Match 2015 Revision UN WPP Country Totals, Revision 11. Palisades, NY: NASA Socioeconomic Data and Applications Center (SEDAC); 2018.

82. Li X, Zhou Y, Zhao M, Zhao X. A harmonized global nighttime light dataset 1992–2018. Scientific Data. 2020;7: 168. doi:10.1038/s41597-020-0510-y

83. Cerdeiro DA, Komaromi A, Liu Y, Saeed M. World Seaborne Trade in Real Time: A Proof of Concept for Building AIS-based Nowcasts from Scratch. International Monetary Fund; 2020. Report No.: WP/20/57.

84. Zhang T, Tian B, Sengupta D, Zhang L, Si Y. Global offshore wind turbine dataset. Scientific Data. 2021;8: 191. doi:10.1038/s41597-021-00982-z

85. Watson RA, Tidd A. Mapping nearly a century and a half of global marine fishing: 1869–2015. Marine Policy. 2018;93: 171–177. doi:10.1016/j.marpol.2018.04.023

86. Tyberghein L, Verbruggen H, Pauly K, Troupin C, Mineur F, De Clerck O. Bio-ORACLE: A global environmental dataset for marine species distribution modelling. Global Ecology and Biogeography. 2012;21: 272–281. doi:10.1111/j.1466-8238.2011.00656.x

87. Assis J, Tyberghein L, Bosch S, Verbruggen H, Serrão EA, De Clerck O. Bio-ORACLE v2.0: Extending marine data layers for bioclimatic modelling. Global Ecology and Biogeography. 2018;27: 277–284. doi:10.1111/geb.12693

88. Global Fishing Watch. Fishing effort. Fleet daily v2 100th degree. 2021;784.

89. Halpern BS, Frazier M, Verstaen J, Rayner P-E, Clawson G, Blanchard JL, et al. The environmental footprint of global food production. Nature Sustainability. 2022; 1–13. doi:10.1038/s41893-022-00965-x

90. Sandwell DT, Gille ST, Smith WHF. Bathymetry from Space:Oceanography, Geophysics, and Climate. Geoscience Professional Services, Bethesda, Maryland; 2002.

91. Rondinini C, Wilson KA, Boitani L, Grantham H, Possingham HP. Tradeoffs of different types of species occurrence data for use in systematic conservation planning: Species data for conservation planning. Ecology Letters. 2006;9: 1136–1145. doi:10.1111/j.1461-0248.2006.00970.x

92. Visalli ME, Best BD, Cabral RB, Cheung WWL, Clark NA, Garilao C, et al. Data-driven approach for highlighting priority areas for protection in marine areas beyond national jurisdiction. Marine Policy. 2020; 103927. doi:10.1016/j.marpol.2020.103927

93. Brito-Morales I, Schoeman DS, Molinos JG, Burrows MT, Klein CJ, Arafeh-Dalmau N, et al. Climate velocity reveals increasing exposure of deep-ocean biodiversity to future warming. Nature Climate Change. 2020; 1–6. doi:10.1038/s41558-020-0773-5

94. Hodapp D, Roca IT, Fiorentino D, Garilao C, Kaschner K, Kesner-Reyes K, et al. Climate change disrupts core habitats of marine species. Global Change Biology. 2023; gcb.16612. doi:10.1111/gcb.16612

95. Ready J, Kaschner K, South AB, Eastwood PD, Rees T, Rius J, et al. Predicting the distributions of marine organisms at the global scale. Ecological Modelling. 2010;221: 467–478. doi:10.1016/j.ecolmodel.2009.10.025

96. Jones MC, Dye SR, Pinnegar JK, Warren R, Cheung WWL. Modelling commercial fish distributions: Prediction and assessment using different approaches. Ecological Modelling. 2012;225: 133–145. doi:10.1016/j.ecolmodel.2011.11.003

97. O'Hara CC, Afflerbach JC, Scarborough C, Kaschner K, Halpern BS. Aligning marine species range data to better serve science and conservation. Guralnick R, editor. PLOS ONE. 2017;12: e0175739. doi:10.1371/journal.pone.0175739

98. Yool A, Shepherd JG, Bryden HL, Oschlies A. Low efficiency of nutrient translocation for enhancing oceanic uptake of carbon dioxide. Journal of Geophysical Research: Oceans. 2009;114. doi:10.1029/2008JC004792
