# Supplementary material for: Cumulative human impacts on global marine fauna highlight risk to biological and functional diversity
Source: PLoS One. 2024 Sep 18;19(9):e0309788. doi: 10.1371/journal.pone.0309788 (PMC11410257; doi:10.1371/journal.pone.0309788)
Supplement: Attachment — Submitted filename: response_to_reviewers.pdf [file pone.0309788.s002.pdf]

Casey O'Hara
National Center for Ecological Analysis and Synthesis
1021 Anacapa St.
Santa Barbara CA 93101
ohara@nceas.ucsb.edu

August 1, 2024

Dear Dr. Pokkathappada,

My coauthors and I thank you and your reviewers for the thorough and thoughtful review of our manuscript, "Cumulative human impacts on global marine fauna highlight risk to biological and functional diversity." We have addressed all the journal requirements and reviewers' comments, and feel that the resulting manuscript is now stronger as a result. Below, we describe the changes we have made (in serif font face) in response to the reviewers' comments (in *italic sans serif face*).

Journal requirements:

1. *Please ensure that your manuscript meets PLOS ONE's style requirements, including those for file naming. The PLOS ONE style templates can be found at https://journals.plos.org/plosone/s/file?id=wjVg/PLOSOne_formatting_sample_main_body.pdf and https://journals.plos.org/plosone/s/file?id=ba62/PLOSOne_formatting_sample_title_authors_affiliations.pdf*

We believe our submission meets all the PLOS ONE style requirements. If there are specific items that we have not met, please provide more detailed information so we can properly address them.

2. *When completing the data availability statement of the submission form, you indicated that you will make your data available on acceptance. We strongly recommend all authors decide on a data sharing plan before acceptance, as the process can be lengthy and hold up publication timelines. Please note that, though access restrictions are acceptable now, your entire data will need to be made freely accessible if your manuscript is accepted for publication. This policy applies to all data except where public deposition would breach compliance with the protocol approved by your research ethics board. If you are unable to adhere to our open data policy, please kindly revise your statement to explain your reasoning and we will seek the editor's input on an exemption. Please be assured that, once you have provided your new statement, the assessment of your exemption will not hold up the peer review process.*

We have uploaded the Github repository containing all code and output data (as a single .zip file) to Figshare, along with a folder containing the primary output rasters as .tif files. This collection has been assigned a DOI (10.6084/m9.figshare.26454106) and we will publish this collection upon acceptance (in case any additional review necessitates further changes).

3. *We note that Figures 2,3,5 and 6 in your submission contain map/satellite images which may be copyrighted.*

All maps figures are original to our analysis, using the code and data generated for our study, and do not contain any copyrighted information. All maps included in figures use land form polygons from the Natural Earth collection (https://www.naturalearthdata.com/), which is public domain and does not require citation (though we have cited the Natural Earth website in the text of the Supporting Methods).

4. *Please include captions for your Supporting Information files at the end of your manuscript, and update any in-text citations to match accordingly. Please see our Supporting Information guidelines for more information:* http://journals.plos.org/plosone/s/supporting-information.

We have provided our Supporting Information as a single PDF containing supporting figures, tables, and methods. We believe our format adheres to the guidelines, and have added a caption for the file at the end of the manuscript (after acknowledgments, before references):

> **"S1 Supporting Information. Supporting figures, tables, methods, and references.** Supporting information includes: Figures S1 – S7, Tables S1 – S4, Supporting Methods, and Supporting References (75-98)."

5. *Please review your reference list to ensure that it is complete and correct. If you have cited papers that have been retracted, please include the rationale for doing so in the manuscript text, or remove these references and replace them with relevant current references. Any changes to the reference list should be mentioned in the rebuttal letter that accompanies your revised manuscript. If you need to cite a retracted article, indicate the article's retracted status in the References list and also include a citation and full reference for the retraction notice.*

We believe our reference list is complete and correct, and are not aware of any cited papers that have been retracted. Several new citations have been added to address the reviewers' comments, and these have been noted in the responses to those comments below.

Reviewer #1

The authors assess threats to marine life by developing, presenting and comparing metrics of human impact as seen through the lens of species, habitat and functional diversity. Bringing together these different analyses at the global level and in a way that allows them to be inter-compared is a new and useful addition to the literature. The paper is well written, interesting and potentially important – I enjoyed reading it and I'm happy to recommend it for publication with a few minor corrections.

We thank the reviewer for the kind words. We appreciate their constructive feedback and hope we have sufficiently addressed their questions and suggestions.

The introduction is concise and informative, placing the study clearly in the context of previous literature. The methods section largely sets out the data sources and calculations in sufficient detail, but it's not clear how the stressor intensities are assigned. It would be helpful to include a few sentences outlining the data sources and a reference to the supporting information. In particular, are the climate stressors defined in relation to present-day conditions or some projected future?

We have added additional information to the methods in the main text describing how stressor intensities are assigned (lines 142-149):

> "Spatial data for stressors relates to some physical quantity related to anthropogenic activity, e.g., brightness of nighttime lights, tonnes of nutrient fertilizer runoff, population density within 25 km of coast, or value of aragonite saturation state. For each stressor, a reference value was determined from the data (typically 99.9th percentile of observed values), a historic baseline (e.g., mean/standard deviation of sea surface temperature from 1985-2015), or ecologically relevant value (e.g., aragonite saturation state of 1) (Table S4). We calculated stressor distributions as a value from 0 (stressor not present) to 1 (stressor at reference point, indicating maximum intensity)."

Line 149: I'm not sure why organic chemical pollutants is the only stressor not included in the study which is mentioned at this point; others are (rightly) mentioned in the discussion, line 470-471.

We thank the reviewer for calling this out. Our present analysis builds on a similar (habitat-focused) analysis in Halpern et al. (2019), which did include organic chemical pollutants. This mention in the methods was originally intended to highlight this difference from the 2019 study. However, it does seem out of place in the methods, so we have removed this specific mention, while retaining the text in the discussion explaining the reason for omitting this stressor.

The various impact scores are created by adding the effects of single stressors and so they cannot include compound effects of more than one stressor acting in combination. This is raised in the discussion, but I suggest that it would be helpful to also acknowledge it in the methods section, with a brief explanation of your reasons for this choice.

The reviewer's suggestion was also echoed by Reviewer 2. We have added text to the methods in the section describing the Cumulative Human Impacts: species method (lines 186-190):

> "Note that this additive model does not account for compound effects of multiple stressors acting in combination, i.e., synergistic or antagonistic effects. Meta-analyses examining two-stressor interactions (Crain et al. 2008; Stockbridge et al. 2020) have observed some non-additive stressor interactions, but additive effects were more commonly reported. Additionally, an additive model requires fewer assumptions, is conceptually tractable, and likely results in more conservative results."

The results section presents a selection of well-designed figures which nicely bring out the main points of this complicated study. I just have a few small points:

Figure 2: the spatial differences don't come out vary clearly in A and B – perhaps the color scale could be adjusted to give more contrast to mid-range values.

We had used a non-linear scale on 2A, B, and C to help visualize differences in the low- to mid-range impact areas, but this deemphasizes the areas where they are particularly high, reducing that contrast. We have changed the scale back to a linear scale but capped the values at the 99.99th percentile (to reduce the influence of outliers on the color scale) and added inset maps showing regions of interest and high variation to the figure. We have updated the analogous habitat map in the supporting figures as well.

Line 284: I'm not sure why quantiles are mentioned, when all the results are presented in terms of quartiles.

We thank the reviewer for pointing this out this inconsistency. We've changed this instance to "quartile."

Lines 327-329: the point about the species method suggesting a higher risk to biodiversity than previous habitat-based analyses seems important, and worth moving to the discussion or mentioning again there.

This point was noted by both reviewers, and we have added text to the abstract, discussion, and concluding paragraph to help highlight this result:

> Abstract (39-42): "Comparing species-level modeled impacts to those based on marine habitats that represent important marine ecosystems, we find that even relatively untouched habitats may still be home to species at elevated risk, and that many species-rich coastal regions may be at greater risk than indicated from habitat-based methods alone."

Discussion (456-461): "Conversely, where a species or functional entity approach predicts higher impacts than the habitat approach, the general structure of the ecosystem may be robust to the stressors present, but the region may be home to one or more highly impacted species, increasing extinction risk and posing greater risk of loss of ecosystem function. Such a pattern is evident in coastal waters across the globe, driven largely by climate impacts (Fig. 4), suggesting greater risk to coastal biodiversity than previously understood from habitat-based methods."

Conclusion (551-555): "Consideration of human impacts across lenses of species, function, and habitat provides a richer understanding of marine ecosystems, and highlights that impacts in species-rich coastal regions may pose greater risk to biodiversity than indicated from habitat-based methods alone."

The discussion is consistent with the results and includes all the points I was hoping would be raised - I have no suggestions for improvement.

We appreciate the reviewer's feedback and we are glad that our discussion has met their expectations.

Supporting information: The equations have not been rendered correctly. This may just be an issue in the review copy, but worth checking.

Looking at the Microsoft Word .docx version that we submitted, with one exception (due to a typo which has been fixed), the equations all appear to be rendered correctly; perhaps as the reviewer suggested this may just be a problem with the review copy. We will verify that the equations are rendered correctly when we resubmit the revised version of the manuscript and supporting information.

Reviewer #2

Summary

The authors present a well-considered study that provides a global overview of species, habitat, and functional entity vulnerability to climate and non-climate anthropogenic stressors. The approach is fairly straightforward, but the methods are missing several key details necessary to understand the nuances associated with the results.

We appreciate the reviewer's feedback, and we feel our modifications to address their concerns have improved the manuscript, particularly the methods.

General Comments/Questions

To understand how the impact of a stressor on a species was calculated (Ii in equation 1) required going to the Supporting Information, then to Butt et al. (2022), then to the Supporting Information in Butt et al. (2022). Is there a way to streamline this pathway?

We thank the reader for this feedback. To help communicate the methods of intersecting species ranges, stressor intensities, and species-stressor vulnerabilities, we have included an additional figure in the Supporting Information to help conceptually explain the process. In addition, we have added a brief note to provide the reader an overview of the process before proceeding into the technical details (lines 85-88):

> "For each species/stressor combination, we intersect the species' range with the spatial distribution of the stressor; impact in each pixel is modeled as the product of stressor intensity and the species' estimated vulnerability to that stressor. (See Fig. S1 for conceptual overview of methods)"

We feel the methods are far clearer thanks to these modifications to address the reviewer's concern.

The assessment builds from the impact of individual stressors on individual species, which is derived from vulnerability of the species to that stressor. It is unclear which traits were used for the sensitivity and adaptive capacity terms within vulnerability in the present study. Considering these values represent the foundation for the rest of the analysis, is there any estimate of the uncertainty of those trait values? How are ranges of values handled, such as intraspecific variation or regional differences?

The full methodology of the trait-based vulnerability framework is detailed in Butt et al. (2022), including detailed answers to the questions noted by the reviewer. However, as the reviewer noted, this is fundamental to the rest of the analysis, and so we have included in our supporting information a table that links traits to the sensitivity and adaptive capacity to each of the stressors included in our study for easier reference.

The original trait-based framework (Butt et al. 2022) incorporated a sensitivity analysis around the numeric scores assigned to different trait values and found that varying the scoring scale (linear/convex/concave) generally resulted in changes in magnitude of vulnerability, but not rank (i.e., lower vulnerability species stayed low, higher stayed high). This does not capture uncertainty in trait values themselves, but it does reflect uncertainty in the translation of trait values to vulnerability scores, which is relevant to our purposes.

Finally, trait values for the trait-based framework (Butt et al. 2022) were elicited from experts and generally provided as a single value for a given species for a given trait. Many traits were categorical and broadly applicable across a taxon (e.g., respiration mechanisms such as gills vs. lungs) and thus intraspecific or regional variation is unlikely. For traits on a continuous scale (e.g., fecundity, body length, extent of occurrence), values were binned into ordinal categories which were generally quite broad, likely able to accommodate intraspecific variation and regional variation.

To address these concerns of the reviewer, we have added caveats and a fuller explanation of the trait methods in the supporting information:

> "The expert-elicited trait values used to calculate vulnerability were given as ordinal or nominal categorical values, generally with a single value attributed to each trait for each species. While a single value does not allow for large-scale intraspecific variability or regional variations in traits, most traits are broadly applicable across a species, genus, or family (e.g., respiration structures of gills vs. lungs vs. diffusion), and for traits representing continuous values (e.g., body size, fecundity, age to maturity), the bins are typically in orders of magnitude, wide enough to accommodate substantial variation without affecting the vulnerability estimate."

Line comments

Line 103. Is the full list of species included in the analysis available anywhere? It is useful to know which species met the necessary criteria for inclusion.

While a full list of more than 21,000 species would overwhelm the supporting information, we have set up a link to a document in the analysis repository that allows a reader to interactively examine the species included. We have included a link to that document within the methods and supporting information.

Lines 113-122. How sensitive is the assessment to the 0.5 probability of occurrence value applied to the AquaMaps? In cases where both IUCN and AquaMaps data were available, how closely did the calculated AquaMaps overlap with the IUCN? Essentially, is it reasonable to consider these two sources similar enough to aggregate them?

We believe that it is reasonable to aggregate the two datasets with appropriate precautions, following suggestions from O'Hara et al. (2017) to improve the alignment between the two. In particular, that paper showed that masking the IUCN range polygons of shallow-water corals to appropriate depths reduces errors of commission, greatly improving the agreement between IUCN and AquaMaps; we have applied this method to all shallow water species in the IUCN set, though we had not cited that study as the source of this method. Finally, per AquaMaps recommended best practice to ensure high quality distribution maps, we omitted AquaMaps species whose maps were generated using fewer than 10 "occurrence cells", i.e., half-degree cells with at least one validated occurrence record, though this detail was not included in our original supporting methods text.

Additionally, the O'Hara et al. 2017 study explored the sensitivity of results (in that case, MPA coverage) to the choice of AquaMaps probability threshold, and our use of a 0.5 probability of occurrence threshold value follows the example of much of the literature within the field (e.g.., Hodapp et al. 2023, Brito-Morales et al. 2021, Visalli et al. 2020, Klein et al. 2015). We have added

additional information and citations for these papers into the supporting methods to help justify our methodology:

> "To determine the range map for each species, we converted gradient probabilities into binary presence/absence using a threshold of ≥0.5 to represent "presence" of a given species, following a common practice in conservation literature (e.g., e.g.., Hodapp et al. 2023, Brito-Morales et al. 2021, Visalli et al. 2020, Klein et al. 2015). Species maps based on fewer than 10 "occurrence cells" (0.5° cells with at least one validated occurrence record) were rejected to ensure quality (Kaschner et al. 2019)."

And:

> "For coastal and neritic species from both datasets, we masked the resulting presence maps to cells with a minimum depth of 200 m or less using GEBCO bathymetry data (Sandwell et al. 2002), and masked using the ocean area raster to exclude non-ocean cells. The depth mask for shallow-water species is an effective method of reducing errors of commission in IUCN range maps to better align them with AquaMaps distribution maps (O'Hara et al. 2017)."

Line 124. Were all species included in the current assessment also assessed in Butt et al. (2022)?

Yes, though as noted below, for the purposes of our assessment, scores were recalculated based on additional trait data and slightly updated methodology.

Line 133. SI Methods – Vulnerability Estimates. $G_i/G_{i,max}$ is an undefined term with no associated text elsewhere in the SI. In the original methodology (Butt et al. 2022), Gi represented general adaptive capacity summed over a series of traits.

This G_i reference in the equation should have been called out in the previous paragraph, but was mistakenly listed as A_i. This has been corrected. The line in the SI now reads:

> "...and life history and population-level traits that affect the population's ability to adapt to or recover from disturbances in general, i.e., general adaptive capacity G_i."

After the equation, the authors present two modifications to the Butt et al. 2022 methodology. They mention body length, fecundity, generation time, temperature tolerances, and depth preferences in addition to extent of occurrence. It is unclear if these traits were used only for those species missing traits in the original Butt et al. (2022) study or if only those traits were used for all species in the present study. Additionally, it is unclear if adaptive capacity values were sourced from Butt et al. (2022) or if they were determined anew in the present study.

All trait categories used in Butt et al. (2022) were used in our updated calculations. For the original Butt et al. study, some traits were provided by our taxon experts at the genus or family level, rather than at the species level. For species in these genera/families, we filled in species-specific values for these traits (body length, fecundity, generation time where available on FishBase/SeaLifeBase, and

thermal tolerances and depth preferences from AquaMaps). These traits are the ones most likely to vary within a taxonomic group. For the remaining traits, values were filled from taxonomic relatives within the original Butt et al. trait data. Taxa not included in Butt et al. (2022) did not have sufficient trait data to calculate vulnerability and so were excluded from our study.

In our updated analysis of trait-based vulnerability, all components of the vulnerability score (sensitivity, specific adaptive capacity, and general adaptive capacity) were recalculated. The exposure term was unchanged.

We have updated the text of the SI methods to help explain this process more clearly. The relevant portion of the updated paragraph (immediately following the vulnerability equation) now reads:

> "First, for species whose traits were provided by taxon experts at genus level or higher rank in the trait set assembled by Butt et al (2022), certain traits (i.e., body length, fecundity, generation time, temperature tolerances, and depth preferences) were filled using species-level data (where available) from FishBase/SealifeBase and AquaMaps, while extent of occurrence was determined from the species' distribution map as described above. Remaining traits were taken as provided by the taxon experts for the original study. Second, rather than calculate sensitivity, adaptive capacity, and vulnerability scores for species groups then impute missing values based on the distribution of those scores (as done in the original study), we first imputed missing trait values based on frequency within the species' taxonomic neighbors, then scored the sensitivity, adaptive capacity, and vulnerability from the combination of species-level traits (from FishBase, SeaLifeBase, and AquaMaps) and those imputed traits."

Additionally, we have included an additional table (Table S3) in the supplement noting which traits were used in calculating vulnerability (breaking into the various components) for each of the stressors included in our study.

Line 133. SI Methods – Vulnerability Estimates. How did the vulnerability scores in this study compare with the vulnerability scores from Butt et al. (2022)? Did the reduced number of traits affect vulnerability scores in a meaningful way?

As noted above, the updated vulnerability estimates used all the same trait categories as the original Butt et al. (2022) study, with differences in the methodology that could result in some variation from the original scores. Additionally, some small discrepancies in the scoring rubric were corrected, which will result in intended deviations from the published scores. Checking the published vulnerability scores against those in our recalculation, we find a mean/sd difference of .0002 +/- .005, with 98% of differences between -0.00009 and 0.00024. We have noted these minor differences in the SI methods:

> "Note that these methodological updates resulted in small differences in the vulnerability scores between the published study and our study: mean/sd difference of .0002 $\pm$ .005, with 98% of differences between -0.00009 and 0.00024."

Lines 138-148. Please describe how stressors were determined to be universal or "per species." UV radiation, sea level rise, light pollution, and ship strike would also seem dependent on position in the water column in a similar manner as bycatch.

The differentiation between universal and per-species stressors was intended to allow for cases where the intensity of impact is not entirely encapsulated by the traits used to estimate vulnerability, but perhaps requires additional species-specific information. The targeted fisheries stressor is highly dependent on species identity (and specifically whether humans consider it a valuable target), while the bycatch stressor is related to the intensity of fishing based on different gear types which we divided into benthic and pelagic zones. Thermal stress is related to the species' preferred thermal envelope, not something that is easily contained in the categorical trait values used to calculate vulnerability. For these cases, we felt a more complex model was required to capture the nuances. We have updated the methods to try to make this more clear (lines 156-161):

> "However, there were also several stressors for which intensity (again ranging from 0 to 1) depends on species-specific information. These stressors include bycatch (dependent on water column position, i.e., benthic, pelagic, or both), biomass removal (dependent on catch that is directly targeting that species), and increase in mean SST (dependent on species thermal tolerance). For these stressors, individual maps were generated for each species (biomass removal, SST rise) or for each water column position category (bycatch)."

Regarding the reviewer's specific examples, it is true that exposure to these stressors would also be dependent on position in the water column. For these we allowed the exposure term E_{ij} to drive the vulnerability to zero if exposure was essentially impossible (e.g., for mesopelagic fish, ship strikes are not a threat). The difference between these and the bycatch stressor is that position in the water column results in changes in intensity, rather than effectively simply turning the stressor on or off. We could probably have treated benthic and pelagic bycatch separately in a similar way using an exposure flag, we just chose a different method.

To clarify the purpose and effect of this exposure flag for future readers, we have updated the text in the methods describing the vulnerability estimates (also related to a related comment below) (lines 134-136):

> "A binary exposure multiplier (zero or one) prevents nonsensical results for certain stressors where exposure is limited to certain depths or ocean zones, e.g., ship strikes will not affect mesopelagic species."

Lines 163-164. How does species-specific thermal stress not apply at the habitat level for things like coral reefs, kelp forests, and seagrass beds?

This was perhaps a poor choice of words to say that thermal stress was "not applicable." Certainly, all species in any given habitat may suffer from thermal stress based on each species' environmental preferences or tolerances. But for the habitat-based impact model, it is impossible to determine a single thermal tolerance that applies broadly across the ecosystem represented by a given habitat,

since that thermal tolerance would necessarily be driven by the community of species present in any particular location (along with complex interactions within the community). We have rephrased this to read (lines 174-175):

> "The species-specific stress of increasing mean SST relative to their thermal tolerance was omitted, as it would not be feasible to determine an analogous habitat-level thermal tolerance."

Lines 168-170. Here, but possibly earlier with the vulnerability estimates, it would be worth mentioning that the exposure term in the vulnerability estimate is a 0 or 1 potential indicator and does not relate to the level or magnitude of exposure. Otherwise, stressor intensity (s sub j) appears to be double counting exposure to a stressor.

Good point - we have included a mention of this earlier in the methods, as noted above:

> "A binary exposure multiplier (zero or one) prevents nonsensical results for certain stressors where exposure is limited to certain depths or ocean zones, e.g., ship strikes will not affect mesopelagic species."

Lines 196-197. How was ph (p sub h) determined?

The original habitat data (for Halpern et al. 2019) was generated at a resolution of ~1 km with values of present/absent for each habitat type. For our 10 km resolution analysis grid, we aggregated these presence/absence values to determine the proportion of each cell that contained each given habitat. We have included such a description in the methods (lines 208-210):

> "...we recreated habitat maps at the same resolution and projection as the species-based analysis, aggregating habitat presence maps at ~1 km resolution to determine proportional habitat representation within each 10 km grid cell."

Lines 276-280. Can you also highlight areas with the least impact?

Great idea - we have added additional information on areas with very low impacts (300-304):

> "Lower cumulative impacts were observed in remote areas where low fishing and shipping activity coincided with relatively low climate stressors (e.g., Southeastern Australia, equatorial Eastern Pacific, Southern Atlantic, Northern Atlantic near Greenland). The very lowest cumulative impacts were observed in areas dominated by permanent or seasonal sea ice (Arctic Ocean, coastal Antarctica)."

Lines 288-295. Possibly in the SI, it would be helpful to see the percent occurrence in each cell of this quartile x quartile matrix presented in Fig 2D in addition to highlighting the cell values of the two most extreme ends of the matrix in the text.

We have included such a matrix in the SI, recreating the legend in Fig. 2D for ease of comparison, but placing percentage occurrence values in each cell of the legend. We have added a sentence to

highlight the most extreme ends of the matrix (the highest two being the hot spots and cool spots already described) (lines 320-322):

> "These hot spots and cool spots represent the two most common instances of impact quartile overlap; conversely, high climate/low non-climate (2.9%) and low climate/high non-climate (3.1%) represent the rarest instances of overlap (Fig. S3)."

Lines 326-329. This seems like a major conclusion from the paper. I would expect to see this mentioned in the abstract and in the concluding paragraphs.

We thank the reviewer for this suggestion, which was also mentioned by Reviewer 1. We have added text to the abstract, discussion, and conclusion to highlight this result:

> Abstract (39-42): "Comparing species-level modeled impacts to those based on marine habitats that represent important marine ecosystems, we find that even relatively untouched habitats may still be home to species at elevated risk, and that many species-rich coastal regions may be at greater risk than indicated from habitat-based methods alone."

> Discussion (456-461): "Conversely, where a species or functional entity approach predicts higher impacts than the habitat approach, the general structure of the ecosystem may be robust to the stressors present, but the region may be home to one or more highly impacted species, increasing extinction risk and posing greater risk of loss of ecosystem function. Such a pattern is evident in coastal waters across the globe, driven largely by climate impacts (Fig. 4), suggesting greater risk to coastal biodiversity than previously understood from habitat-based methods."

> Conclusion (551-555): "Consideration of human impacts across lenses of species, function, and habitat provides a richer understanding of marine ecosystems, and highlights that impacts in species-rich coastal regions may pose greater risk to biodiversity than indicated from habitat-based methods alone."

Lines 411-413. This is a methodological decision that could probably be better explained/justified. Given that these estimates may be synergistic, why use an additive approach instead of one that could include those potential synergies?

This echoes a comment by Reviewer 1 as well. We chose to use a simple additive approach since a more complex model that incorporates synergistic or antagonistic factors would require mechanistic understandings of such interactions to generate and parameterize a sensible model. Meta-analyses of multiple-stressor interactions (e.g., Crain et al. 2008, Stockbridge et al 2020) typically are limited to stressor pairs, and show additive effects (or fail to show significant synergistic effects) for many if not most stressor combinations studied. We have added some text to the methods (rather than here in the discussion) to justify this additive choice (lines 186-190):

> "Note that this additive model does not account for compound effects of multiple stressors acting in combination, i.e., synergistic or antagonistic effects. Meta-analyses examining two-

> stressor interactions (Crain et al. 2008; Stockbridge et al. 2020) have observed some non-additive stressor interactions, but additive effects were more commonly reported. Additionally, an additive model requires fewer assumptions, is conceptually tractable, and likely results in more conservative results."

Note also, the reviewer points out that the text here in lines 411-413 is duplicative of text in lines 474-477. We have removed the explanation in lines 411-413, leaving in place the explanation in lines 474-477.

Lines 465-471. Are there any examples where locally important stressors that were not included due to data coverage may have outweighed the impact measured by those stressors included in the current study?

We appreciate the reviewer's insightful question. We can definitely think of contexts in which this could be the case, and have added a note to reflect this (lines 502-506):

> "Important and/or emerging stressors such as marine plastics, noise pollution, persistent organic chemical pollutants, human-driven sedimentation, and seabed mining should be priorities for future cumulative human impact models, especially as there may be localized contexts in which the impact of such stressors may outweigh those included in our study."

Lines 474-477. This is repetitive of lines 411-413. Including with this paragraph does highlight that this decision may result in more conservative results, but additional rationale for the decisions would provide necessary context.

As noted earlier, we have added some text to the methods to explain and justify our choice of an additive model, and removed the redundant text from lines 411-413.

Lines 516-520. I can readily see how this approach can inform decision-makers about locations for conservation but it's less clear how this can inform strategies for conservation. Can the authors provide examples of conservation strategies beyond MPAs that this work would inform given spatial resolution, species included, and climate/non-climate stressors considered? Within these 4x4 matrices presented in figures 2D, 3A/B, and 5A/B, is there a prioritization scheme for MPAs or other area-specific management strategies?

"Strategies for conservation" could mean a variety of things. For example, within MPAs, there are multiple strategies for protecting marine species and ecosystems, such as no-take marine reserves, partially protected areas, etc. Another interpretation for 'strategies for conservation' could refer to what management action is required to best mitigate a threat to species/ecosystem, such as MPAs versus non-MPA actions (restoration of a habitat, modification of fishing gear to reduce bycatch, reduce land-based run-off, etc). Although we believe we have covered this throughout the discussion (paragraph 2 and last paragraph), to help make this more concrete, in the concluding paragraph, we have provided an example of a non-MPA conservation action/strategy that could be considered based on our results (lines 546-548):

> "...necessitating conservation action to mitigate non-climate stressors (e.g., reduction of land-based runoff) to allow for improved ecosystem resilience to climate change."

Additionally, we agree that our reference to "strategies for conservation" in the final sentence was not clear so we have revised it entirely to focus on conservation action (lines 553-555):

> "Our data can be used with socioeconomic information to help prioritize effective, economically efficient, and socially equitable conservation actions to best benefit nature and people."

We thank you for your consideration and effort in reviewing our manuscript. We believe our revisions have addressed all comments thoroughly and look forward to any additional feedback.

Sincerely, on behalf of all coauthors,

Dr. Casey C. O'Hara

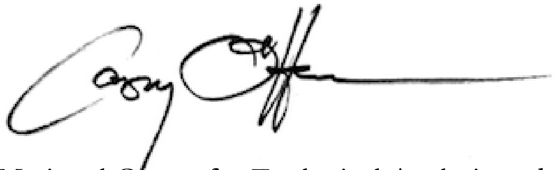

National Center for Ecological Analysis and Synthesis
Santa Barbara CA 93101
ohara@nceas.ucsb.edu
